# Nipple-Sparing Mastectomy and Immediate Breast Reconstruction with da Vinci SP Surgical System: The First Case in Japan

**DOI:** 10.70352/scrj.cr.24-0187

**Published:** 2025-08-19

**Authors:** Yuko Kijima, Munetsugu Hirata, Yumika Nakazawa, Kazuya Shimmura, Naoki Hayashi, Ryunosuke Kijima, Yoshikazu Inoue, Hiroshi Nishioka, Ichiro Uyama

**Affiliations:** 1 Department of Breast Surgery, Fujita Health University, School of Medicine, Toyoake, Aichi, Japan; 2 Department of Plastic Surgery, Fujita Health University, School of Medicine, Toyoake, Aichi, Japan; 3 Advanced Robotic and Endoscopic Surgery, Fujita Health University, School of Medicine, Toyoake, Aichi, Japan

**Keywords:** breast cancer, da Vinci SP, robotic surgery, nipple-sparing mastectomy, breast reconstruction

## Abstract

**INTRODUCTION:**

Nipple-sparing mastectomy (NSM) has been increasingly used therapeutically for breast cancer patients in whom the nipple-areolar complex is not involved, being associated with better esthetic results and QOL than skin-sparing mastectomy. Robotic nipple-sparing mastectomies (R-NSM) using da Vinci SP surgical system (Intuitive Surgical, Sunnyvale, CA, USA) with immediate breast reconstruction (IBR) has been reported as a suitable surgical treatment for early breast cancers. We present a patient who underwent R-NSM and IBR, with excellent results. To our knowledge, this is the 1st reported case of R-NSM and IBR using the da Vinci SP surgical system in Japan.

**CASE PRESENTATION:**

We performed R-NSM for a 41-year-old Japanese woman with cTisN0M0 breast cancer. NSM and IBR to place a tissue expander (TE) into the post-pectoral pocket were performed using the da Vinci SP surgical system with the double bipolar method.

**CONCLUSIONS:**

This is the 1st reported case of R-NSM using da Vinci SP surgical system in Japan.

## INTRODUCTION

The recently released da Vinci SP surgical system (Intuitive Surgical, Sunnyvale, CA, USA) is a true single-port platform with all instruments entering one single port.^1,2)^ We present a patient who underwent robotic nipple-sparing mastectomy (R-NSM) and immediate breast reconstruction (IBR), with excellent results. To date, there have been only a few reports worldwide on R-NSM using the da Vinci SP system, and none have been reported in Japan.^1,3–6)^ This is the 1st reported case of R-NSM and IBR using the da Vinci SP surgical system in Japan.

## CASE PRESENTATION

### Patient

**Fig. 1:**
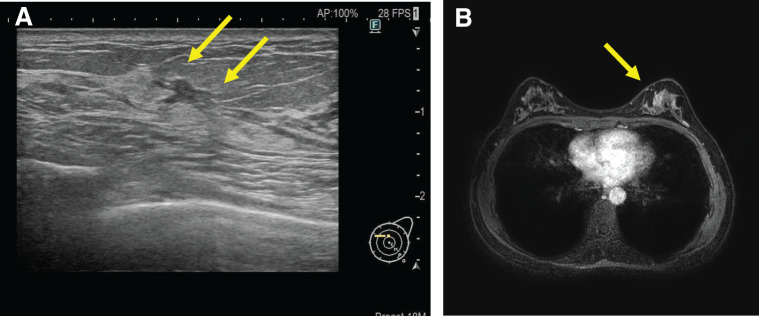
Images of the patient’s preoperative evaluation. (**A**) Ultrasonography of the left breast showed a 2.0 cm non-mass lesion with intraductal calcification without nipple involvement. (**B**) MRI showed suspicions of clumped enhancement in the upper-inner quadrant area of the left breast.

**Fig. 2:**
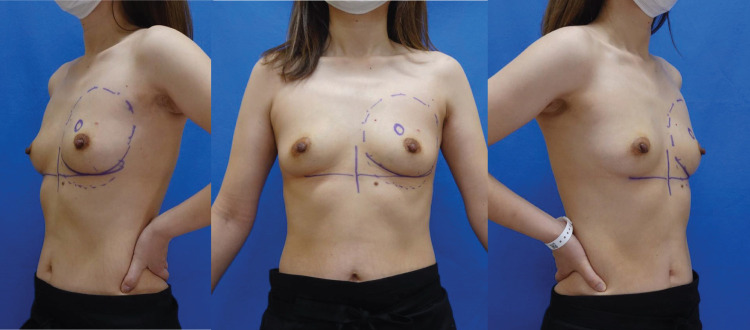
Preoperative findings in a 41-year-old woman with a slim body and non-ptotic breasts. The cancer lesion was located in the inner-upper area of the breast.

A 41-year-old Japanese woman noticed a breast mass located in the inner-upper quadrant area of the left breast. Preoperative imaging investigations included mammography breast ultrasound imaging, breast MRI, CT, and FDG-PET (**Fig. 1**). She was diagnosed with TisN0M0 Stage 0 breast cancer based on the TNM classification. On a genetic test, no mutations were detected in BRCA1/2. She was slim and her breasts were non-ptotic (**Fig. 2**). The patient’s height was 155.5 cm, weight was 45.3 kg, and BMI was calculated to be 18.7. She had no history of drinking or smoking. We planned R-NSMs to be performed by a surgical team (Y.K., M.H., and I.U.), and IBR by a plastic surgery team (Y.I. and H.N.). Informed consent was obtained from the patient for R-NSM and IBR, involving the insertion of tissue expander (TE) under the major pectoral muscle. Our institutional ethical committee approved of the R-NSM procedures. Written informed consent was also obtained from the patient for the publication of this case report.

### Surgical procedure

Initially, sentinel lymph node biopsy was performed by the surgical group through a longitudinal skin incision along the mid-axillary line. Before the incision, the breast borders were marked using indigo carmine blue dye. The length of the incision was 3.5 cm. Sentinel lymph nodes were biopsied via axillary incision directly, revealing no metastases. Then, we dissected the outer part of the breast tissue from the major pectoral muscle using scissors and bipolar coagulation by direct vision. Subsequently, an access port of the da Vinci SP surgical system was placed on the incision. Through this access port, the SP surgical system with one camera and three arms was inserted in the outer area of the breast. The camera stood in the cobra position for the medial prepectral dissection. Until the blue dye was observed along the edge of the breast tissue, dissection between the breast tissue and pectoral major muscle was performed using fenestrated bipolar forceps and Maryland forceps, with counter-retracing. We undermined the breast tissue from the major pectoral muscle completely. The fascia of the major pectoral muscle was observed, while that of the cancerous area was resected. The next step of undermining the breast tissue from the skin was directly performed using scissors and electric devices, in the same manner as for the posterior procedure. Before the robotic procedure we infected 100 mL of tumescent solution into the subcutaneous layer using a Vessel needle to prevent bleeding during tunneling, according to a previous report.^4)^ This procedure was performed under ultrasonographical monitoring to reach the surface of the breast tissue. Following this, tunneling using Metzenbaum scissors in the subcutaneous flap was performed. After docking, the robotic procedure for the superficial breast tissue was started at 10 mm on the outer apart from the nipple–areolar complex. We dissected the subcutaneous tissues between the tunnels while maintaining the proper thickness of the flap. The surgical margin just under the nipple was examined intraoperatively to assess whether cancer involvement was positive. Histological examination revealed that infiltration was negative. We utilized the “double bipolar method,” a technique we developed, where Maryland bipolar forceps in the surgeon's right hand are used for tissue dissection, and fenestrated bipolar forceps in the left hand are employed for hemostasis. This method causes less distant electrical burn injury and smoke compared with monopolar electric cautery.^7)^

**Fig. 3:**
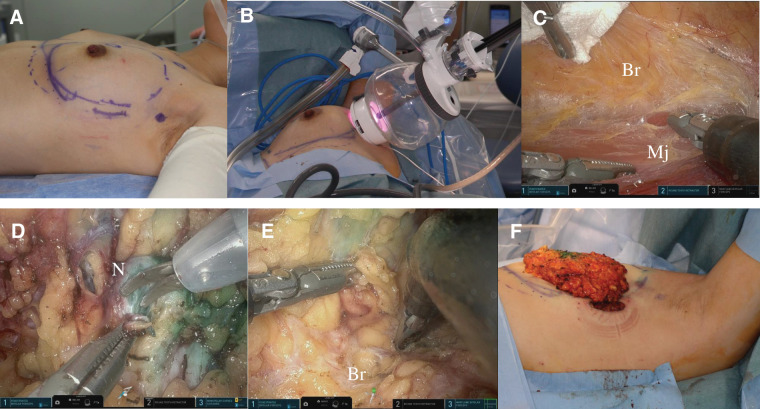
Oncological procedure. (**A**) A 35-mm longitudinal skin incision on the mid-axillary line. (**B**) An access port of the da Vinci SP surgical system placed on the skin incision. (**C**) Dissection of the breast tissue from major pectoral muscle. (**D**) Surgical margin of the nipple examined intraoperatively. (**E**) Dissection of the breast tissue from skin. (**F**) Resected breast tissue via the 35-mm skin incision. Br, breast tissue; Mj, major pectoral muscle; N, nipple

After superficial dissection, peripheral dissection along with the blue dye proceeded from the lateral to an inner area of the breast. We resected 141 g of breast tissue without extending the 35-mm primary incision (**Fig. 3**).

### Breast reconstructive procedure

**Fig. 4:**
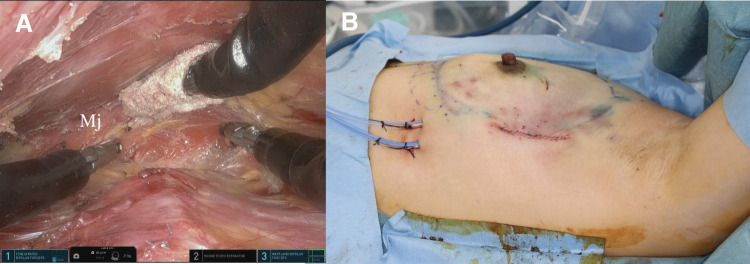
Reconstructive procedure. (**A**) The cranial and inner edges of the pectoral pocket approached by the robotic procedure. (**B**) Findings at the end of surgery. Mj, major pectoral muscle

A pectoral pocket to insert the TE was prepared by direct and robotic approaches. The outer and cranial parts of the pocket including the pectoral major muscle and a part of the anterior serratus muscle were elevated by direct vision, and the lower and inner parts of the post-pectoral area were elevated by a robotic approach. We inserted a 300-cc TE according to the resected tissue size. After the insertion of TE, two drainage systems were inserted in the pre- and post-pectoral areas, respectively (**Fig. 4**).

The oncological time was 178 min, the console time was 66 min, and the reconstructive time was 74 min. The amount of intra-operating bleeding was 33 cc. The length of hospital stay after R-NSM was 9 days.

**Fig. 5:**
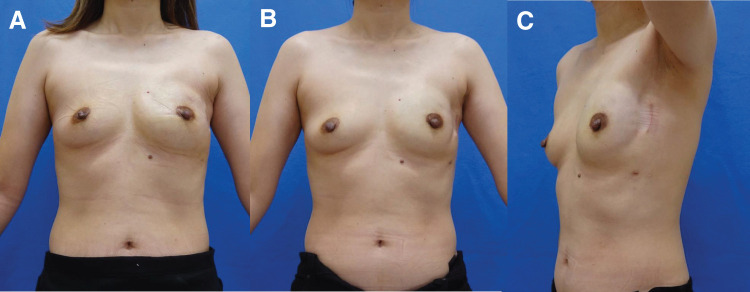
Postoperative findings. (**A**, **B**) Seven postoperative months after R-NSM. (**C**) A postoperative month after exchange of TE to SBI. R-NSM, robotic nipple-sparing mastectomy; SBI, silicon breast implant; TE, tissue expander

There was no postoperative complication due to either R-NSM or the reconstruction procedure. The surgical scar was inconspicuous, and the cosmetic result was excellent (**Fig. 5**).

### Post R-NSM and IBR procedure

During the 7 months after R-NSM, a total of 200 mL of normal saline was injected. Delayed breast reconstruction was performed, exchanging from TE to a silicon breast implant (SBI) (165 cc).

## DISCUSSION

NSM was initially reserved for the prophylactic treatment of women with a high risk of developing breast cancer.^8)^ Now, NSM is being increasingly applied therapeutically for breast cancer patients without nipple–areolar complex involvement.^9–12)^ NSM is associated with better esthetic results and QOL than skin-sparing mastectomy.^13,14)^

R-NSM using da Vinci Xi system was introduced in 2015, and has several advantages, including flexible robotic arms with a high-resolution 3D camera, ergonomic console, compensation for hand-shaking, and detailed movement of robotic arms with high-level degrees of freedom. Through a very small axillary wound, the da Vinci surgical platform can be introduced to perform R-NSM, followed by IBR.^15)^ This surgical technique has been reported to facilitate promising surgical and cosmetic outcomes.^16,17)^

The consensus statement on robotic mastectomy referred to the indications, contraindications, technical considerations, patient counseling, outcome measures, indicators, and training and learning curve assessment.^18)^ According to this statement, we selected this case to be indicative for a 1st case performed in R-NSM; patients’ indications included breast size, breast shape, and nipple position; oncological indications were DCIS, spread of intraductal component, and no cancer involvement of nipple and lymph node.

The 1st description of R-NSM with the da Vinci SP surgical system was reported by Joo et al. in 2022. They concluded that da Vinci SP was the most appropriate platform for carrying out mastectomy and breast reconstruction through a small single incision.^3)^ In our case, we dissected breast tissue from the major pectoral muscle by direct vision, and then dissected using a robotic procedure. Subsequently, we dissected breast tissue from the skin with non-robotic scissors followed by robotic dissection to avoid skin injury via a 3.5-cm skin incision on the mid-axillary line. As a result, we could avoid skin injury and preserve good flow to the nipple.

A major type of breast reconstruction following R-NSM using da Vinci SP was one-stage reconstruction.^3,4,6)^ According to these reports, IBRs were performed via autologous reconstruction in 63 patients, implant covered in acellular dermal matrix in the prepectoral pocket in 92 patients, and TE in 32 patients, respectively.^1,3–6)^ Out of 32 cases reconstructed using TE, Farr et al. reported IBR using covered TE with acellular dermal matrix insertion in the prepectoral pocket in 20 patients.^1)^ In 12 cases reconstructed using TE, the details were not mentioned.^4,6)^ Joo et al. reported that the reason they selected subpectoral TE insertion was that the patient wanted augmented breasts. They mentioned that implant insertions into the same plane for both breasts would be more comfortable to the patient.^3)^

In this case, we introduced double bipolar method previously reported by Katsuno et al. for robotic total mesorectal excision in patients with rectal cancer. They concluded that the double bipolar method was more useful than the single bipolar method, in which a pair of monopolar curved scissors were used.^7)^ In the previous reports referring R-NSM for breast cancer patients, no discussion about the instruments to undermine breast tissue from the skin and the major pectoral muscle exists. Although the use of the double bipolar method for R-NSM has not been reported, we introduced this method for our 1st case. We undermined the breast tissue from the skin and the major pectoral muscle, and made a subpectoral pocket for insertion of TE without any injury. We were convinced that the double bipolar method should be useful for R-NSM as well as for robotic surgery of gastrointestinal tissue.

As of the end of 2024, R-NSM has yet to be covered by insurance in Japan. While robotic-assisted surgeries are becoming more widely used in other medical fields, their application to breast cancer surgery remains limited in terms of indications and insurance coverage. Regarding the da Vinci surgical system, although certain components have received regulatory approval, it has not yet become a standard treatment option for breast reconstruction or breast cancer surgery. Challenges such as high implementation costs, the need for surgeons to develop expertise, accumulation of clinical evidence, and adjustments to the insurance system are factors contributing to this situation. In the future, with further accumulation of evidence and potential reforms in the healthcare system, robotic-assisted surgeries may see broader applications in breast cancer treatment. Continued attention to developments in this area is essential.

## CONCLUSIONS

We successfully performed Japan’s 1st NSM and IBR using the da Vinci SP system. This could become a popular surgical option for early breast cancer patients.

## DECLARATIONS

### Funding

We have no funding source.

### Authors’ contributions

All authors were involved in performing this clinical procedure.

Y.K., M.H., I.U., H.N., and Y.I performed surgery.

K.S., Y.N., R.K., and N.H. contributed to postoperative procedures and researched the previous published articles.

All authors have reviewed and approved the manuscript submission.

### Availability of data and materials

The datasets used and/or analyzed during the current study are available from the corresponding author on reasonable request.

### Ethics approval and consent to participate

This study was approved by the Institutional Review Board of Fujita Health University Hospital (23-14), and the patient provided their written informed consent for inclusion in this study.

### Consent for publication

Consent for publication has been obtained.

### Competing interests

We declare no financial relationships or other interests associated with this manuscript, which might be construed as constituting a conflict of interest.
